# Graft-Versus-Host Disease Prophylaxis with Antithymocyte Globulin in Patients Receiving Stem Cell Transplantation from Unrelated Donors: An Observational Retrospective Single-Center Study

**DOI:** 10.3390/cancers15102761

**Published:** 2023-05-15

**Authors:** Mariella Lo Schirico, Roberto Passera, Jessica Gill, Chiara Dellacasa, Irene Dogliotti, Luisa Giaccone, Sofia Zompi, Alessandro Busca

**Affiliations:** 1 Department of Medicine (DIMED), Hematology and Clinical Immunology Section, Padua University School of Medicine, 35121 Padova, Italy; 2 Onco Hematology, Department of Oncology, Veneto Institute of Oncology IOV-IRCCS, 31033 Castelfranco Veneto, Italy; 3 Department of Medical Sciences, Division of Nuclear Medicine, University of Torino, Corso AM Dogliotti 18, 10126 Torino, Italy; 4 Department of Oncology, SSD Trapianto Allogenico di Cellule Staminali, Azienda Ospedaliera Universitaria (A.O.U.) Città della Salute e della Scienza di Torino, 10126 Torino, Italy; 5 Department of Molecular Biotechnology and Health Sciences, Division of Hematology, University of Torino, 10124 Torino, Italy

**Keywords:** bone marrow transplantation, unrelated donor (URD), antilymphocyte serum (ATG), Graft-versus-Host disease (GvHD)

## Abstract

**Simple Summary:**

Graft-versus-host disease (GVHD) remains a main cause of morbidity and mortality in patients receiving allogeneic hematopoietic stem cell transplantation. Among GVHD prophylaxis regimens, antilymphocyte serum (ATG/ATLG) has been widely used in both unrelated donor transplant and with HLA–identical sibling donation from peripheral blood stem cells, at the cost of increased opportunistic infections, as well as relapses for delayed immune reconstitution. Moreover, there are differences in dosage and formulation with ATG which make difficult to compare results. We performed a retrospective single-center analysis on a cohort of 226 patients receiving ATG at a fixed dose of 5 mg/kg in unrelated donor transplants. GVHD’s cumulative incidence was 29.9% and 29.8%, for acute and chronic GVHD, respectively. We recorded relapse incidence and infection rates in line with other GVHD prophylaxis regimens. Thus, we suggest that this low dose of ATG could be used as an effective GVHD prophylaxis without a significant worsening of other transplant outcomes.

**Abstract:**

Graft-versus-host disease (GVHD) is one of the most important complications of allogeneic hematopoietic stem cell transplantation. Rabbit antilymphocyte serum (ATG/ATLG) is recommended for GVHD prophylaxis, while its appropriate dosing is debated. We performed a retrospective single-center study to examine the outcome of patients receiving ATG at the dose of 5 mg/kg as GVHD prophylaxis for unrelated donor (URD) HSCT. We collected data from all consecutive adult patients with hematological malignancies who had undergone allogeneic HSCT from URDs at the Stem Cell Transplant Center of the Città della Salute e della Scienza Hospital of Torino between July 2008 and July 2021. The primary aim was to ascertain the cumulative incidence (CI) for acute GVHD (aGVHD) and chronic GVHD (cGVHD); the secondary aim was to ascertain the CI for NRM (Non-Relapse Mortality) and RI (Relapse Incidence), as well the overall survival (OS) and infection incidence within 30 days of transplantation. We included in the analysis 226 patients who collectively underwent 231 HSCTs. The CI of grade II–IV aGVHD was found to be 29.9%, while that of moderate to severe cGVHD was 29.8%. The CI of NRM recorded at 1, 2, and 3 years after transplant was 18.2%, 19.6%, and 20.2%, respectively. The CI of RI at 1, 2, and 3 years from transplant was recorded to be 17.8%, 21.0%, and 21.6%, respectively. The median follow-up was 56 months, while the median OS for the whole cohort was not established; the OS at 1, 3, and 5 years from transplant was 69.6%, 59.3%, and 57.2%, respectively. We registered 88 bacteremias in 82/231 patients (35.5%), while invasive fungal infections occurred in 12/231 patients (5.2%). Our study suggests that the use of ATG at 5 mg/kg is highly effective in limiting the occurrence of both aGVHD and cGVHD, ensuring a low NRM, RI, and infection incidence.

## 1. Introduction

Allogeneic hematopoietic stem cell transplantation (HSCT) is the only curative therapy for many high-risk hematological malignant and non-malignant diseases [1], however, its efficacy is limited by a wide spectrum of complications, including infections, graft failure and, most importantly, both acute and chronic graft-versus-host disease (GVHD) [2,3]. 

Rabbit antilymphocyte serum is widely used in GVHD prophylaxis, together with a calcineurin inhibitor and either methotrexate or mycophenolate mofetil [4]. Two formulations are currently available: antithymocyte globulin (ATG, Sanofi Genzyme, Cambridge, MA, USA), originated from rabbit immunization with human thymocytes, and anti-T-lymphocyte globulin (ATLG, Neovii, Rapperswil, Switzerland), originated from immunization with the human Jurkat T-cell line. Given their different dosages, the two products are not interchangeable, and there is no advice regarding the choice of the brand [5].

The use of ATG/ATLG is particularly recommended in patients receiving grafts from matched or mismatched unrelated donors (URD) and patients receiving peripheral blood (PB) HSCT from HLA–identical sibling donors [5]. Due to its effect both on donor and recipient T-lymphocytes, ATG/ATLG is effective both in reducing the incidence and severity of GVHD and in preventing graft failure; however, it can be associated with delayed immune reconstitution and an increased risk of infectious complications [6,7]. In addition, the most appropriate dosing strategy of ATG/ATLG is a matter of debate, with a broad range of doses reported in literature [5]. In addition, scattered studies suggest ATG/ATLG dosing should be tailored based on absolute lymphocyte count (ALC), rather than on the patient’s body weight [8,9]. This new concept is based on the hypothesis that ALC is the cellular target, and its use might better reflect ATG/ATLG clearance, leading to optimal exposure.

In this single-center observational retrospective study, we investigated the outcome of patients receiving ATG at the dose of 5 mg/kg as GVHD prophylaxis for URD HSCT.

## 2. Patients and Methods

### 2.1. Study Design and Procedures

We retrospectively collected data from all consecutive adult patients with hematological malignancies who had undergone allogeneic HSCT from URD at the Stem Cell Transplant Center of the Città della Salute e della Scienza Hospital of Torino between July 2008 and July 2021. We included only patients receiving BM/PB grafts from URD and ATG dosed at 5 mg/kg as part of GVHD prophylaxis. Patients receiving multiple allo-HSCTs during the study period were censored at the time of their second HSCT, and data were collected independently for each single transplant. All URD underwent typing at HLA–A, HLA–B, HLA–C, HLA–DRB1, and HLA–DQB1.

Patients received either myeloablative (MAC) or reduced intensity (RIC) conditioning regimens based on the patient’s age and clinical conditions, in accordance with the hematopoietic cell transplantation-specific comorbidity index (HCT-CI). MAC regimens include, in the majority of cases, busulfan-containing regimens (total dose > 6.4 mg/kg, intravenously) associated with thiotepa and/or fludarabine; by contrast, acute lymphoblastic leukemia patients received total body irradiation and cyclophosphamide. In patients over 60 years of age and/or in presence of pre-transplant comorbidities, RIC regimens were usually preferred, including single dose of thiotepa associated with busulfan (total dose ≤ 6.4 mg/mg/kg, intravenously) and fludarabine (or, alternatively, fludarabine), in addition to cyclophosphamide, and low dose total body irradiation (200 Gray). 

The cytogenetic risk was calculated according to the disease-specific scoring risk (e.g., ELN 2017 for AML, R-IPSS for MDS, or R-ISS for MM).

ATG was administered at a dose of 2.5 mg/kg on days −3 and −2, or 0.5 mg/kg on day −3, 2 mg/kg on day −2, and 2.5 mg/kg on day −1. Chlorphenamine, paracetamol, and methylprednisolone were given as a premedication. GVHD prophylaxis also included cyclosporin A (CSA) and a short course of methotrexate. All patients received antiviral prophylaxis with acyclovir 800 mg BID, and antifungal prophylaxis with fluconazole 200 mg BID. Patients received antibacterial prophylaxis with levofloxacin at 500 mg/day. Letermovir was given as cytomegalovirus (CMV) reactivation prophylaxis in CMV-positive recipients since 2019. ALC was calculated on the first day of ATG administration.

GVHD incidence and severity were defined according to standard criteria, and on clinical and histological bases [10,11].

Infections were recorded within 30 days of transplantation. Bacterial infections were defined as bloodstream infections (BSIs) when documented according to blood culture. Invasive fungal infections (IFIs) were considered only if proven to be probable, in accordance with the European Organization for Research and Treatment of Cancer (EORTC)/Mycoses Study Group (MSG) criteria [12].

All clinical data were obtained from a retrospective review of medical charts, and the date for the last follow-up was set as 30 April 2022.

Written consent for both transplant procedures and the use of medical records for research purposes was obtained from all patients. Due to the retrospective observational nature of this research, and in accordance with Italian law (Agenzia Italiana del Farmaco-AIFA, Guidelines for observational studies, 20 March 2008), no formal approval from the local Institutional Review Board/Independent Ethics Committee was needed.

### 2.2. Statistical Analysis

The primary end goals were to ascertain the cumulative incidence (CI) for acute GVHD (aGVHD) and chronic GVHD (cGVHD) (main events); their competing events were either the relapse/death without aGVHD in the first 120 days from transplant, or the relapse/death without cGVHD in the first 2 years from transplant, respectively. The secondary goals were to ascertain the CI for Nonrelapse Mortality (NRM) and the RI (Relapse Incidence) as well as the overall survival (OS) and the infection incidence within the day +30 post-transplantation. As for NRM, the main event was the death without relapse, while its competing event was the relapse; likewise, for RI, the reverse. All aGVHD, cGVHD, NRM, and RI cumulative incidence curves were compared using the Gray test, while the Fine–Gray test was applied for the competing risks regression model.

As for the OS, it was defined as the time from transplant to death from any cause; OS curves were estimated using the Kaplan–Meier method and compared with the log-rank test. Additionally, the OS was analyzed using the uni- and multivariate Cox proportional hazards model, comparing the covariates’ effect with the Wald test, for which aGVHD and cGVHD occurrence (as well disease status at transplant) were treated as time-dependent variables.

Graft and relapse free survival (GRFS) was estimated according to Holtan et al. [13]; we therefore considered grade II-III-IV aGVHD, moderate-severe cGVHD, relapse and death as events.

The following covariates were tested as potential risk factors in all models: recipient age (≥60 vs. 40–60 vs. <40 years), recipient gender (male vs. female), diagnosis (AML/ALL vs. other hematological malignancies), cytogenetic risk (intermediate/high vs. low), recipient and donor CMV status (positive vs. negative), disease status at transplant (advanced vs. early disease), Sorror Comorbidity Index (HCT-CI, ≥3 vs. 0–2), HLA match grade (mismatched vs. matched), stem cell source (bone marrow vs. peripheral blood), conditioning regimen (reduced intensity vs. myeloablative), median absolute lymphocyte count before ATG dose (≥200 vs. <200 × 10^6^/L), median CD34 doses infused/recipient weight (≥7.5 × 10^6^/kg vs. <7.5 × 10^6^/kg), median CD3 doses infused/recipient weight (≥2.7 × 10^8^/kg vs. <2.7 × 10^8^/kg), median duration of neutropenia (≥18 vs. <17 days), acute (grade II–IV vs. 0–I) and chronic GVHD (moderate/severe vs. no and mild) occurrence and year of transplant (2018–2021 vs. 2013–2017 vs. 2008–2012).

Patient characteristics were estimated using the Fisher’s exact test for categorical variables and the Mann-Whitney test for continuous ones, which were reported as medians [interquartile range (IQR)]. All *p*-values were obtained using the two-sided exact method, at the conventional 5% significance level. Data were analyzed as of November 2022 by R 4.2.1 (R Foundation for Statistical Computing, Vienna-A, http://www.R-project.org (accessed on 30 November 2022)).

## 3. Results

### 3.1. Patients’ Characteristics

During the study period, 226 patients underwent 231 HSCT and have been included in the analysis: 226 patients had one HSCT, while the remaining five had two. The main patient characteristics at transplant are listed in Table 1. Median age at the time of transplant was 51 years (IQR 43–60), and 126 were male (54.5%). Underlying diseases were acute leukemia (65.3%), Hodgkin and non-Hodgkin lymphomas (18.2%), chronic myeloproliferative diseases (8.7%), and other hematological malignancies (7.8%). Around 80% of patients were grafted in complete remission (117/179, 65.4% in first CR; 27/179, 15.1% in second CR) while 35/179 (19.6%) in advanced stage of disease. The cytogenetic risk was classified as intermediate/high for 96/149 subjects (64.4%), while one third of the patients had an HCT-CI ≥ 3. The main graft source was peripheral blood in 94.4% of the patients (218/231). Overall, 199 patients (86.1%) received a myeloablative conditioning regimen. Patients received HSCT from 10/10 or 9/10 HLA-matched URD in 80% of the cases. 

### 3.2. Cumulative Incidence of aGVHD and cGVHD

Overall, grade II–IV aGVHD occurred in 73/231 patients (raw incidence, 31.6%) in the first 120 days following transplant, resulting in a cumulative incidence of 29.9% (Table 2, Supplementary Figure S1); its competing event (relapse/death without aGVHD) was 13.9%. In univariate analyses, an increasing recipient age (from 24.4% to 40.4%), an advanced disease status at transplant (38.7% vs. 26.5%) and the year of transplant (progressively decreasing from 34.0% to 26.7%) were factors marginally associated with the risk of aGVHD. Thus, no multivariate competing risk regression model was estimated for grade II–IV aGVHD occurrence. 

Moderate to severe cGVHD occurred in 60/191 patients (raw incidence, 31.4%) in the first 2 years from transplant, resulting in a cumulative incidence of 29.8%; the competing event (relapse/death without cGVHD) was 19.4% (Table 2, Supplementary Figure S2). In univariate models, HLA mismatching (*p* = 0.003) and grade II–IV aGVHD (*p* = 0.007) were significantly associated to the risk of cGVHD, while the year of transplant and recipient age showed a marginal trend. A multivariate competing risk regression model for cGVHD (Table 3) confirmed both the role of HLA mismatching as risk factor for cGVHD (SDHR [subdistribution hazard ratio] 2.14, *p* = 0.008) and grade II–IV aGVHD (SDHR 1.86, *p* = 0.021).

### 3.3. Cumulative Incidence of NRM and RI

NRM occurred in 50/231 patients (raw incidence, 21.6%), resulting in a cumulative incidence at 1, 2 and 3 years following transplant of 18.2%, 19.6%, and 20.2%, respectively (Supplementary Table S1, Supplementary Figure S3). 

Univariate analyses showed that an increased recipient age, a HCT-CI ≥3, recipient CMV positive status, a duration of neutropenia ≥ 18 days and a RIC regimen (34.6% vs. 15.6%) were all significantly associated with NRM. In the multivariate competing risk regression model for NRM (Table 3), recipient age (SDHR 3.52, *p* = 0.002) and duration of neutropenia (SDHR 2.55, *p* = 0.047) were confirmed as statistically significant covariates. 

Relapse (RI) occurred in 53/231 patients (raw incidence, 22.9%) leading to a cumulative incidence at 1, 2 and 3 years from transplant of 17.8%, 21.0%, and 21.6%, respectively (Supplementary Table S1, Supplementary Figure S3). 

The univariate models showed that HCT-CI ≥3 (27.6% vs. 14.5%), intermediate/high cytogenetic risk (27.3% vs. 9.6%), grade II–IV aGVHD (13.1% vs. 25.0%) and moderate/severe cGVHD (6.2% vs. 27.0%) were factors significantly associated with a higher cumulative incidence of relapse. The multivariate competing risk regression model for RI (Table 3) confirmed the prognostic role of cytogenetics (SDHR 3.00, *p* = 0.028) and moderate/severe cGVHD (SDHR 0.08, *p* = 0.013).

### 3.4. Overall Survival

The median follow-up for surviving patients was 56 (IQR 30–81) months. Overall, 98 patients died (42.4%), 48 for relapsed disease, and 50 for transplant-related complications; in particular, 28 for infection, eight for GVHD, three for second tumors, two for graft failure and nine for other toxicities (four cardiac, three neurological, and two hepatic). 

Notably, the median OS for the whole cohort was not reached; the cumulative OS at 1, 3 and 5 years from transplant was 69.6%, 59.3%, and 57.2%, respectively (Supplementary Figure S4). 

Intermediate/high cytogenetic risk (*p* = 0.039), CMV positive recipients (*p* = 0.031), HCT-CI ≥ 3 (*p* < 0.001), RIC conditioning regimen (*p* = 0.039), moderate/severe cGVHD (*p* < 0.001), and the year of transplant (*p* = 0.005) were factors significantly associated with OS in the univariate analysis series. In the multivariate Cox proportional hazards model, HCT-CI (HR 3.09, *p* < 0.001) and moderate/severe cGVHD (HR 0.12, *p* = 0.001) were associated with a worse OS (Supplementary Table S2).

**Figure 1:**
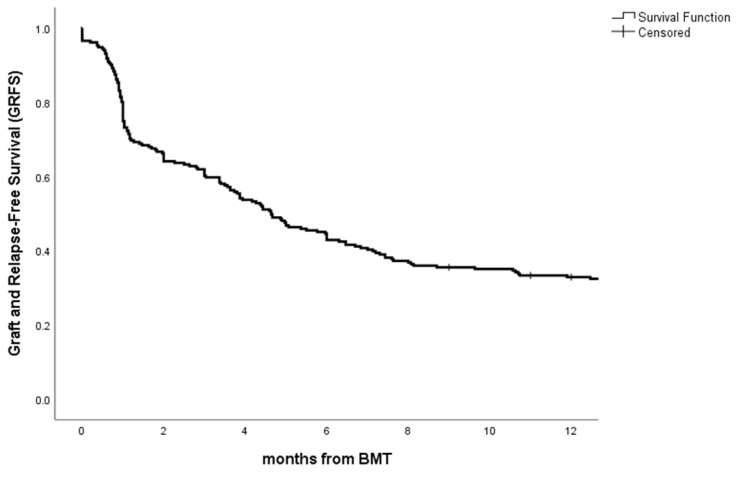
Graft- and relapse-free survival for the whole cohort.

GRFS at 12 months was 32.9%, with a median GRFS of 4.63 months (Figure 1). 

### 3.5. Infections

**Figure 2:**
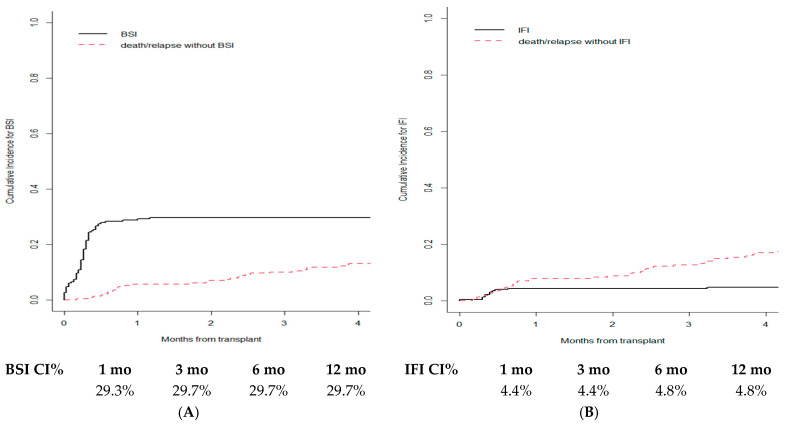
Cumulative incidence for BSI (**A**) and IFI (**B**) in whole cohort.

We registered 88 BSIs in 82/231 patients (35.5%, as six patients had two episodes of bacteremia). Out of 88 BSIs, 39 (44.3%) were caused by Gram-positive and 41 (46.6%) by Gram-negative bacteria, while eight (9%) were polymicrobial. Cumulative incidence for BSI was 29.3% at 1 month following transplantation, reaching a plateau of 29.7% beyond third month until first year (Figure 2A). 

Proven or probable IFI occurred in 12/231 patients (5.2%), with a mortality rate of 58.3% (7/12), which was significantly higher than the rate observed in patients without IFI (41.1%; 90/219) (*p* = 0.049). Cumulative incidence for IFI was 4.4% at both 1 and 3 months after transplant, and then reached a plateau of 4.8% at 6 and 12 months (Figure 2B). 

**Figure 3:**
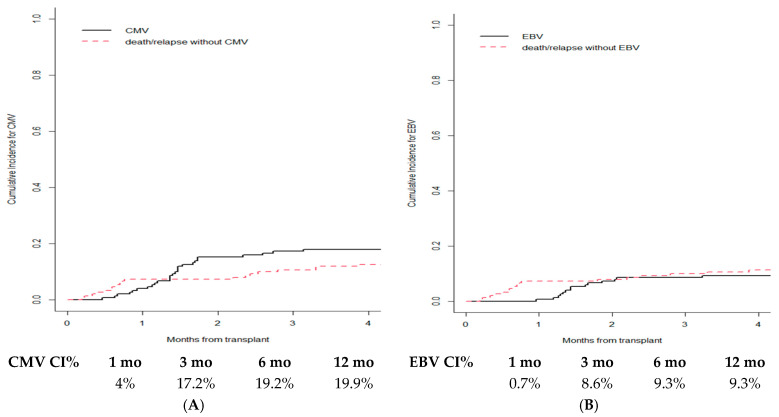
Cumulative incidence for CMV (**A**) and EBV (**B**) in whole cohort.

A total of 33 CMV reactivation were described (33/231, 14%), with a cumulative incidence of 4.0% at 1 month after HSCT which rises to 17.2% at 3 months (Figure 3A). Most patients who reactivated CMV did not take a letermovir prophylaxis; 29/33 no letermovir vs. 4/33 letermovir, for the CMV-CI was 4.9% vs. 0% after 1 month, respectively, and the difference thinned over years (*p*-value = 0.221) (Supplementary Figure S5). 

EBV reactivation occurred in 18 patients (18/231, 8%), with a cumulative incidence of 0.7% at 1 month after transplant, which increased to 8.6% at 3 months (Figure 3B). 

## 4. Discussion

Both aGVHD and cGVHD remain a major complication in patients undergoing HSCT from URD. There has been a surge of evidence in favor of a beneficial effect of ATG combined with conventional GVHD prophylaxis, such as calcineurin inhibitors and MTX. Accordingly, an international expert panel provided consensus-based recommendations [5] emphasizing that ATG/ATLG is particularly effective in those patients receiving grafts from matched or mismatched URD. Their recommended doses are 4.5–7.5 mg/kg for ATG and 60 mg for ATLG. 

The present study retrospectively analyzed a large cohort of adult patients receiving both grafts from URD and a homogeneous GVHD prophylaxis based on ATG. Our study suggests that the use of ATG at 5 mg/kg is highly effective for limiting the occurrence of both aGVHD and cGVHD. 

In a previous multi-center study, we showed that ATG doses higher than 5 mg/Kg in patients receiving HSCT from URD were associated with worse GRFS and infection-related mortality [14]. Interestingly, the rate of grade II–IV aGVHD was superimposable between the previous and the current study (28.6% vs. 29.9%, respectively) as were the OS (56% vs. 57%, respectively) and NRM (21.5% vs. 20%, respectively). A slightly higher rate of cGVHD has been reported in this study (29% vs. 17.4%, respectively), however the number of patients receiving PBSC grafts (86% vs. 94% respectively) might explain these findings. By contrast, infectious complications seem to have had a marginal impact on the outcome of patients that were included in this study, possibly reflecting a better management of infections during most recent years.

Four main randomized trials comparing ATG/ATLG-based GVHD prophylaxis vs. standard GVHD prophylaxis with CSA–MTX in URD recipients have been published.

Bacigalupo et al. [15] compared two different doses of ATG (7.5 mg/kg and 15 mg/kg) combined with CSA–MTX vs. CSA–MTX alone in patients receiving BM from URD; the incidence of grade II–IV aGVHD was 69% when ATG was administered at 7.5 mg/kg and 37% for a dosing of 15 mg/kg, while the incidence of moderate-severe cGVHD was 38% and 41%, respectively. Taken as a whole, a significant reduction of both aGVHD and cGVHD was demonstrated in the ATG group as compared to those who received CSA–MTX only; however, no difference in terms of relapse rate, NRM, or OS has been documented, due to an excess of infections in patients who were given higher doses of ATG. 

Walker et al. [16] evaluated ATG at a low dose of 4.5 mg/kg in association with CSA–MTX vs. CSA–MTX alone in URD HSCT recipients mostly grafted with PBSC: final results of this multicenter phase 3 trial showed that the cumulative incidence of cGVHD was significantly reduced in ATG group vs. standard GVHD prophylaxis group (26% vs. 41% respectively, *p* = 0.032), as well as OS (70% vs. 53% respectively, *p* = 0.017), while relapse incidence and NRM were superimposable. 

Two studies [8,17] on the use of ATLG 60 mg in URD recipients showed a significant reduction of grade II–IV aGVHD and cGVHD in patients who received ATLG, compared with those receiving prophylaxis with CSA–MTX only. Therefore, despite the retrospective nature of our study, the results compare favorably to those reported in literature. It should be underscored that the high degree of HLA-matching in our donor–recipient pairs (80% 9/10 or 10/10) might have contributed to dampen the severity of GVHD; nevertheless, our analysis showed that HLA-matching had no impact on aGVHD.

Notwithstanding that the use of ATG is associated with delayed immune reconstitution (and therefore with high risk of infections, and eventually a high rate of NRM), our study showed that the rates of BSI and IFI were consistently low; 35% and 5%, respectively. These findings compare favorably with those reported by Finke et al. [17] on the use of ATLG (62% of BSI, and 33% of IFI). Likewise, we observed a 14% incidence of CMV infection, remarkably lower than the rates reported in the prospective trials of Bacigalupo et al. [15] (78–83%) and Soiffer et al. [8] (62%), and even in the retrospective studies of Kuriyama et al. [18] (83%) and Othman et al. [19] (41%). It is worthwhile recalling that our findings might be explained, at least in part, by the use of letermovir as a CMV prophylaxis in 30 patients. 

Similarly, the 18% CI of NRM after 1 year was substantially low, especially considering that one third of the patients had high HCT-CI.

The immunosuppressive activity of ATG, mitigating the Graft versus Leukemia (GvL) effect, may result in a higher risk of relapses of the underlying disease. Bacigalupo et al. [15] reported that patients receiving the higher dose of ATG had a 1-year RI of 36%, compared with 18% in the cohort of patients not receiving ATG. In our study, the 1-year RI was 17.8%: if we consider that only 35.6% of patients had a low-risk disease, we can assume that the dose of ATG might have preserved a GVL effect.

Prior studies suggested tailoring ATG dosing according to ALC rather than the patient’s weight [8,9]: to tackle this issue, we investigated whether different values of ALC could turn into a different outcome. In our study, ALC at the time of ATG administration had no impact on acute and chronic GVHD, NRM, RI, and OS, suggesting that we need additional data to confirm that this approach may be adopted to individualize ATG dosing.

We recognize several limitations inherent to our study. First, this was an observational retrospective single-center study; second, our patients received a fixed dose of ATG, making it impossible to compare whether different dosages might be even more effective; third, novel regimens of GVHD prophylaxis have been tested in URD transplants. 

Recently, several studies analyzed the use of post-transplant cyclophosphamide (PT/Cy) for GVHD prophylaxis, not only in the haploidentical setting but also in recipients of HSCT from MUD [20,21,22]. Results from a retrospective study [22] in MUD HSCT, showed that grade III–IV aGVHD (8% vs. 9%, *p* = 0.5) and cGVHD (18% vs. 19%, *p* = 0.5) were superimposable between patients receiving PT/Cy and ATG-based prophylaxis, but PFS was improved (57% vs. 48%, *p* = 0.01) and NRM was lower (13% vs. 23%, *p* = 0.02) among patients in the PT/Cy cohort, possibly due to a lower risk of viral infections and related death. On the other hand, no statistically significant differences in OS were noted between the two groups.

## 5. Conclusions

In conclusion, our study indicated that ATG at 5 mg/Kg allowed a good control of GVHD, maintaining a low incidence of NRM, RI and infections. Prospective trials evaluating the efficacy of PTCy vs. ATG in patients receiving HSCT from unrelated donors are ongoing, in order to identify the best strategy to minimize the risk of GVHD, and improve survival and quality of life of patients undergoing this procedure.

## Figures and Tables

**Table 1:** Main patients’ and transplants’ characteristics of the whole cohort.

Characteristics.	All Patients
Number of patients/transplants	226/231
Age at transplant, median (IQR), years	51 (43–60)
*Gender* Male Female	126 (54.5%) 105 (45.5%)
*Underlying disease* AML/MDS ALL HL/NHL MPN/LMMC other	123 (53.2%) 28 (12.1%) 42 (18.2%) 20 (8.7%) 18 (7.8%)
*HCT-CI* Low/intermediate (0–2) High (>3)	119 (68.4%) 55 (31.6%)
*Cytogenetics* Low risk Intermediate/High risk	53 (35.6%) 96 (64.4%)
*Disease status at transplant* CR1 CR2 PD	117 (65.3%) 27 (15.1%) 35 (19.6%)
*CMV Donor status* negative positive	128 (57.4%) 95 (42.6%)
*CMV Recipient status* negative positive	67 (30.3%) 154 (69.7%)
*Stem cell source* PBSC BM	218 (94.4%) 13 (5.6%)
Number of Ly at ATG administration, median (IQR), ×10^6^/L	200 (90–570)
Number of CD34+ cells infused, median (IQR), ×10^6^/kg	7.5 (5.8–10.6)
Number of CD3+ cells infused, median (IQR), ×10^8^/kg	2.7 (2.0–3.5)
Duration of neutropenia, median (IQR), days	17 (15–21)
*Conditioning regimen* MAC RIC	199 (86.1%) 32 (13.9%)
*Recipient–donor HLA matched alleles* 10/10 and 9/10 ≤8/10	179 (79.6%) 46 (20.4%)
*Year of transplant* 2008–2012 2013–2017 2018–2021	47 (20.3%) 98 (42.4%) 86 (37.3%)
*Acute GvHD* 0–I II–IV	158 (68.4%) 73 (31.6%)
*Chronic GvHD* absent/mild moderate/severe	131 (68.6%) 60 (31.4%)
*Nonrelapse mortality and relapse incidence* alive without relapse dead without relapse relapsed	128 (55.5%) 50 (21.6%) 53 (22.9%)
*Overall Survival* alive dead	133 (57.6%) 98 (42.4%)

Abbreviations: *AML* acute myeloid leukemia; *MDS* myelodysplastic syndromes; *ALL* acute lymphoblastic leukemia; *HL* Hodgkin lymphoma; *NHL* non-Hodgkin lymphoma; *MPN* myeloproliferative neoplasms; *LMMC* chronic myelomonocytic leukemia; *HCT-CI* hematopoietic cell transplantation-comorbidity index; *CR1* first complete remission; *CR2* second complete remission; *PD* progressive disease; *CMV* cytomegalovirus; *PBSC* peripheral blood stem cell; *BM* bone marrow; *IQR* inter quartile range; *MAC* myeloablative conditioning; *RIC* reduced intensity conditioning; *ATG* antithymocyte globulin; *GvHD* graft versus host disease.

**Table 2:** Cumulative incidence for aGVHD (at d120) and cGVHD (at 2 years), overall and stratified by the main risk factors.

Characteristics	aGVHD at d120	*p*-Value *	cGVHD at 2 Years	*p*-Value *
Overall cumulative incidence	29.9%	-	29.8%	-
Age at transplant <40 years 40–60 years >60 years	24.4% 27.1% 40.4%	0.150	28.9% 27.3% 37.2%	0.473
*Gender* Male Female	29.5% 30.2%	0.719	30.1% 29.5%	0.834
*Underlying disease* AML/ALL other	27.5% 31.1%	0.799	29.5% 30.6%	0.918
*HCT-CI* 0–2 ≥3	31.9% 27.3%	0.715	32.1% 22.0%	0.177
*Cytogenetics* Low risk Interm/high	41.5% 24.0%	0.018	29.8% 28.8%	0.872
*Disease status at transplant* CR1 other	26.5% 38.7%	0.071	29.4% 34.7%	0.614
*CMV Donor status* negative positive	30.5% 28.4%	0.395	27.5% 32.5%	0.609
*CMV Recipient status* negative positive	26.9% 32.5%	0.469	30.5% 30.2%	0.920
*Stem cell source* PBSC BM	30.3% 23.1%	0.444	18.2% 30.6%	0.350
Number of Ly at ATG administration, under median over median	26.7% 33.6%	0.172	28.3% 31.6%	0.671
Number of CD34+ cells infused under median over median	33.0% 28.7%	0.355	30.0% 28.6%	0.926
Number of CD3+ cells infused under median over median	32.7% 31.9%	0.974	25.0% 35.0%	0.278
Duration of neutropenia, under median over median	34.8% 28.7%	0.188	28.7% 30.7%	0.606
*Conditioning regimen* MAC RIC	31.2% 21.9%	0.570	30.4% 26.1%	0.647
*Recipient–donor HLA matched alleles* 10/10 & 9/10 ≤8/10	28.5% 37.0%	0.230	25.0% 51.4%	0.003
*Year of transplant* 2008–2012 2013–2017 2018–2021	34.0% 30.6% 26.7%	0.634	25.0% 37.2% 23.3%	0.071
*Acute GvHD* 0–I II–IV	-	-	23.1% 41.4%	0.007

* Comparisons calculated using the Gray test.

**Table 3:** Multivariate competing risks regressions for cGVHD, NRM, and RI.

Characteristics	cGVHD SDHR (95%CI)	cGVHD *p*-Value *	NRM SDHR (95%CI)	NRM *p*-Value *	RI SDHR (95%CI)	RI *p*-Value *
*Age at transplant* (>60 vs. 40–60 vs. <40 years)			3.52 (1.58–7.86)	0.002		
*HCT-CI* (≥3 vs. 0–2)			1.32 (0.50–3.46)	0.570	1.87 (0.90–3.89)	0.092
*Cytogenetics* (interm/high vs. low risk)					3.00 (1.12–8.02)	0.028
*CMV Recipient status* (positive vs. negative)			1.09 (0.37–3.22)	0.880		
*Duration of neutropenia* (over vs. under median)			2.55 (1.01–6.40)	0.047		
*Conditioning regimen* (RIC vs. MAC)			1.13 (0.34–3.75)	0.850		
*Recipient-donor HLA matched alleles* (≤8/10 vs. ≥9/10 vs)	2.14 (1.22–3.77)	0.008				
*Acute GvHD* (II–IV vs. 0–I)	1.86 (1.10–3.13)	0.021			0.97 (0.40–2.37)	0.950
*Chronic GvHD* (moderate/severe vs. absent/mild)					0.08 (0.01–0.59)	0.013

* Comparisons calculated using the Fine–Gray test.

## Data Availability

The data presented in this study are available in this article.

## Supplementary Materials

The following supporting information can be downloaded at: https://www.mdpi.com/article/10.3390/cancers15102761/s1, Figure S1: Cumulative incidence of aGVHD and death/relapse without aGVHD; Figure S2: Cumulative incidence of cGVHD and death/relapse without cGVHD; Figure S3: Cumulative incidence of NRM and RI. Figure S4: Overall Survival for the whole cohort. Figure S5: Cumulative incidence for CMV reactivation, stratified by letermovir prophylaxis. Table S1. Cumulative incidence for NRM (at 1 year) and RI (at 2 years), overall and stratified by the main risk factors. Table S2. Univariate and multivariate Cox proportional hazard models for Overall Survival after allogeneic HSCT.
