# Supplementary material for: Graft-Versus-Host Disease Prophylaxis with Antithymocyte Globulin in Patients Receiving Stem Cell Transplantation from Unrelated Donors: An Observational Retrospective Single-Center Study
Source: Cancers (Basel). 2023 May 15;15(10):2761. doi: 10.3390/cancers15102761 (PMC10216361; doi:10.3390/cancers15102761)
Supplement: Supplementary file 1 [file cancers-15-02761-s001.zip › cancers-2253864-supplementary.pdf]

Supplementary material

Figure S1. Cumulative incidence of aGVHD and death/relapse without aGVHD.

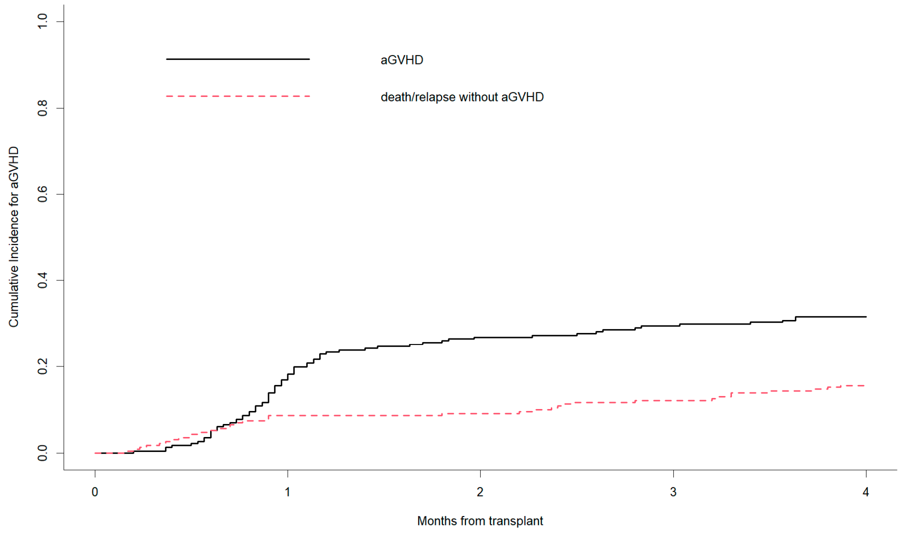

Figure S2. Cumulative incidence of cGVHD and death/relapse without cGVHD.

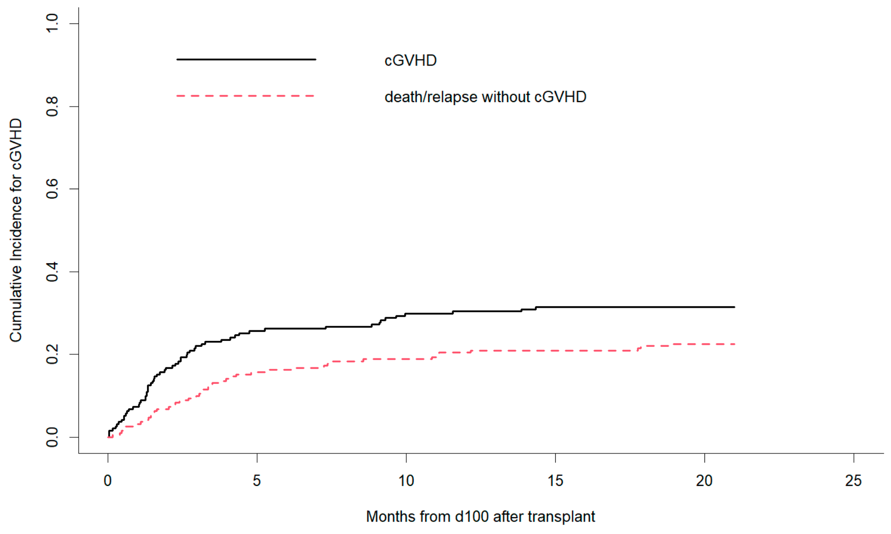

Figure S3. Cumulative incidence of NRM and RI.

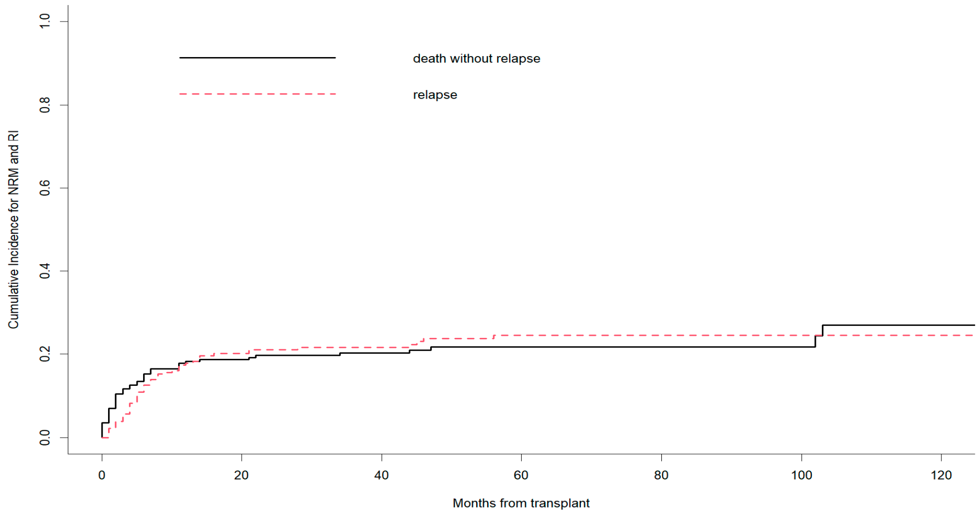

Figure S4. Overall Survival for the whole cohort.

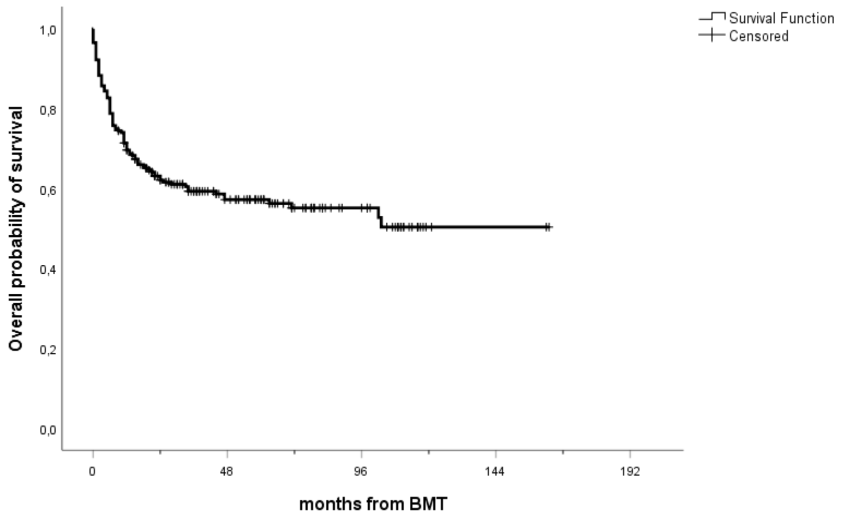

Figure S5. Cumulative incidence for CMV reactivation, stratified by letermovir prophylaxis.

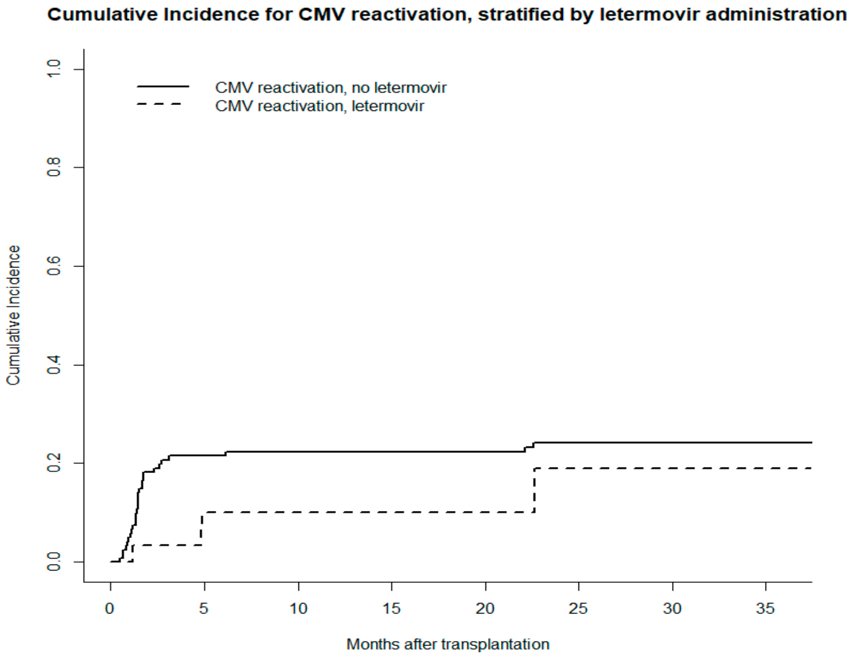

CMV CI %	1 mo	3 mo	6 mo	12 mo	24 mo	36 mo
**No letermovir** 29 events	4.9%	20.7%	21.5%	22.3%	24.2%	24.2%
**Letermovir** 4 events	0	3.3%	10%	10%	18.9%	18.9%

Table S1. Cumulative incidence for NRM (at 1 year) and RI (at 2 years), overall and stratified by the main risk factors.

Characteristics	NRM at 1 yr	p-value*	RI at 2 yrs	p-value*
Overall cumulative incidence	18.2%	-	21.0%	-
Age at transplant				
<40 yrs	6.7%	<0.001	27.2%	0.116
40-60 yrs	14.8%		22.8%	
>60 yrs	35.1%		12.3%	
Gender				
Male	19.0%	0.570	24.0%	0.447
Female	17.2%		17.4%	
Underlying disease				
AML/ALL	17.5%	0.489	22.9%	0.501
other	18.6%		20.0%	
HCT-CI				
0-2	6.7%	0.007	14.5%	0.021
≥3	25.5%		27.6%	
Cytogenetics				
Low risk	15.1%	0.996	9.6%	0.005
Interm/High	16.7%		27.3%	
Disease status at transplant				
CR1	16.3%	0.949	17.4%	0.239
other	16.2%		26.0%	
CMV Donor status				
negative	19.5%	0.444	19.6%	0.292
positive	14.8%		23.6%	
CMV Recipient status				
negative	12.0%	0.042	21.4%	0.953
positive	20.8%		19.6%	
Stem cell source				
PBSC	38.5%	0.194	21.3%	0.829
BM	17.0%		15.4%	
Number of Ly at ATG administration,				
under median	19.9%	0.522	22.9%	0.998
over median	15.9%		19.6%	
Number of CD34+ cells infused				
under median	14.3%	0.171	19.8%	0.611
over median	20.4%		22.5%	
Number of CD3+ cells infused				
under median	15.8%	0.800	21.8%	0.669
over median	15.0%		19.5%	
Duration of neutropenia,		0.057		0.997

under median	9.0%		20.8%	
over median	16.8%		22.0%	
Conditioning regimen				
MAC	15.6%	0.012	21.3%	0.674
RIC	34.6%		18.9%	
Recipient-donor HLA matched alleles				
10/10 & 9/10	17.3%	0.683	22.7%	0.131
≤8/10	19.6%		15.2%	
Year of transplant				
2008-2012	31.9%	0.031	23.4%	0.595
2013-2017	15.3%		21.5%	
2018-2021	14.0%		18.7%	
Acute GvHD				
0-I	17.6%	0.236	25.0%	0.021
II-IV	19.5%		13.1%	
Chronic GvHD				
absent/mild	22.4%	0.136	27.0%	<0.001
moderate/severe	7.6%		6.2%	

* Comparisons calculated using Gray-test.

Table S2. Univariate and multivariate Cox proportional hazard models for Overall Survival after allogeneic HSCT

	**Univariate models**		**Multivariate model**	
	HR (95% CI)	**p-value**	HR (95%CI)	**p-value**
Age	1.30 (0.73-	0.080	1.30 (0.73-	0.127
(40-60 vs. <40 years)	2.31)	0.369	2.31)	0.157
(>60 vs. <40 years)	1.93 (1.04-	0.038	1.93 (1.04-	0.976
	3.59)		3.59)	
Gender (male vs. female)	1.30 (0.87-1.95)	0.201	-	-
Underlying disease (AML/ALL vs. other)	1.06 (0.69-1.62)	0.795	-	-
HCT-CI (≥3 vs. 0-2)	3.11 (1.86-5.21)	<0.001	3.09 (1.70-5.63)	<0.001
Cytogenetics (interm/high vs. low risk)	1.80 (1.02-3.20)	0.044	1.46 (0.77-2.79)	0.248
Disease status at transplant (other vs. CR1)*	1.27 (0.70-2.30)	0.434	-	-
HLA (mismatched vs. matched)	0.84 (0.50-1.40)	0.493	-	-
CMV donor status (positive vs. negative)	1.07 (0.71-1.61)	0.750	-	-
CMV recipient status (positive vs. negative)	1.69 (1.04-2.76)	0.035	1.00 (0.45-2.25)	0.994
Year of transplant	0.51 (0.32-	0.007	2.10 (0.50-	0.288
(2013-2017 vs. 2008-2012)	0.82)	0.005	8.79)	0.309
(2018-2021 vs. 2008-2012)	0.49 (0.29-	0.007	1.32 (0.30-	0.712
	0.82)		5.71)	
Graft source (PBSC vs. BM)	0.76 (0.35-1.64)	0.487	-	-
Number of Ly at ATG administration (over vs. under median), 10^6/l	0.86 (0.58-1.29)	0.470	-	-
Number of CD34+ cells infused (over vs. under median), 10^6/kg	1.34 (0.89-2.02)	0.169	-	-
Number of CD3+ cells infused (over vs. under median), 10^8/kg	0.89 (0.57-1.40)	0.892	-	-
Duration of neutropenia (over vs. under median), days	1.36 (0.88-2.11)	0.172	-	-

Conditioning (RIC vs. MAC)	1.71 (1.02-2.90)	0.044	1.15 (0.48-2.76)	0.749
Acute GvHD II-IV (yes vs. no) *	0.66 (0.39-1.13)	0.130	-	-
Chronic GvHD moderate-severe (yes vs. no) *	0.19 (0.09-0.40)	<0.001	0.12 (0.04-0.43)	0.001
